# Supplementary material for: The effect of workforce diversity on organizational performance with the mediation role of workplace ethics: Empirical evidence from food and beverage industry
Source: PLoS One. 2024 Jul 18;19(7):e0297765. doi: 10.1371/journal.pone.0297765 (PMC11257269; doi:10.1371/journal.pone.0297765)
Supplement: S1 Appendix — (DOCX) [file pone.0297765.s001.docx]

# **Appendix**

| Appendix Table 1 :Stratified Sampling of Food and Beverage Employees | | | | | |
| --- | --- | --- | --- | --- | --- |
| **Strata** | **Industrial Group/STRATA** | **Number*** | **Percentage+** | **n_f_** | **n_e_+** |
|  | Production, processing and preserving of meat,  fruit and vegetables | 3475 | 5.39 | 14 | 22 |
|  | Manufacture of edible, vegetable and animal oils and fats | 1310 | 2.03 | 5 | 8 |
|  | Manufacture of dairy products | 987 | 1.53 | 4 | 6 |
|  | Manufacture of grain mill products | 9318 | 14.46 | 37 | 58 |
|  | Manufacture of prepared animal feeds | 1989 | 3.09 | 8 | 12 |
|  | Manufacture of bakery products | 10662 | 16.55 | 42 | 66 |
|  | Manufacture of sugar and sugar confectionery | 9565 | 14.84 | 38 | 59 |
|  | Manufacture of macaroni and spaghetti | 2962 | 4.60 | 12 | 18 |
|  | Manufacture of food products Not Elsewhere Classified (N.E.C.) | 3759 | 5.83 | 15 | 23 |
|  | Distilling, rectifying and blending of spirits | 3811 | 5.91 | 15 | 24 |
|  | Manufacture of wines | 576 | 0.90 | 2 | 4 |
|  | Manufacture of malt liquors and malt | 5545 | 8.61 | 22 | 34 |
|  | Manufacture of soft drinks & production of mineral waters | 10476 | 16.26 | 41 | 65 |
|  | ***Total*** | 64435 | ***100*** | 254 | ***400*** |

n_f_=Random sampled firms n_e_=Random sampled employees

| Appendix Table 2:Rotated Component Matrix^a^ | | | | | | |
| --- | --- | --- | --- | --- | --- | --- |
|  | Component | | | | | |
|  | PD | OP | RD | IN | IS | DE |
| pd1 | .839 |  |  |  |  |  |
| pd2 | .827 |  |  |  |  |  |
| pd3 | .793 |  |  |  |  |  |
| pd4 | .835 |  |  |  |  |  |
| pd5 | .828 |  |  |  |  |  |
| pd6 | .801 |  |  |  |  |  |
| pd7 | .755 |  |  |  |  |  |
| pd8 |  |  |  |  |  |  |
| pd9 |  |  |  |  |  |  |
| op1 |  | .762 |  |  |  |  |
| op2 |  | .775 |  |  |  |  |
| op3 |  | .767 |  |  |  |  |
| op4 |  | .759 |  |  |  |  |
| op5 |  | .796 |  |  |  |  |
| op6 |  | .723 |  |  |  |  |
| rd1 |  |  | .770 |  |  |  |
| rd2 |  |  | .692 |  |  |  |
| rd3 |  |  | .815 |  |  |  |
| rd4 |  |  | .619 |  |  |  |
| rd5 |  |  | .760 |  |  |  |
| rd6 |  |  | .773 |  |  |  |
| rd7 |  |  |  |  |  |  |
| in1 |  |  |  | .821 |  |  |
| in2 |  |  |  | .751 |  |  |
| in3 |  |  |  | .751 |  |  |
| in4 |  |  |  | .818 |  |  |
| in5 |  |  |  | .753 |  |  |
| is1 |  |  |  |  | .805 |  |
| is2 |  |  |  |  | .761 |  |
| is3 |  |  |  |  | .785 |  |
| is4 |  |  |  |  | .603 |  |
| de1 |  |  |  |  |  | .653 |
| de2 |  |  |  |  |  | .726 |
| de3 |  |  |  |  |  | .656 |
| Extraction Method: Principal Component Analysis.  Rotation Method: Varimax with Kaiser Normalization. | | | | | | |
| a. Rotation converged in 6 iterations. | | | | | | |

## **Appendix II: Questionnaire**

***Part I: The following items are set to measure the prevalence of Employee Perceived Diversity specific to target firms.*** *The replies should range from* ***1: Strongly disagree to 5: Strongly agree.*** *This instrument has been adopted from the work of* Kundu and Mor (2017).

| ***Item*** | ***1*** | ***2*** | ***3*** | ***4*** | ***5*** |
| --- | --- | --- | --- | --- | --- |
| ***Perceived diversity management*** | | | | | |
| 1. Diverse employees bring new perspectives to the organization |  |  |  |  |  |
| 1. Diversity management taps the contributions of all the employees |  |  |  |  |  |
| 1. All employees benefit from effective diversity management |  |  |  |  |  |
| 1. Information for working effectively in a diverse workforce is welcomed |  |  |  |  |  |
| 1. Perceptions of discrimination by diverse employees necessitate attention to diversity management |  |  |  |  |  |
| 1. I work with people who are different from me |  |  |  |  |  |
| 1. Responsibility of top management in valuing diversity |  |  |  |  |  |
| 1. Gender diversity is important |  |  |  |  |  |
| 1. Organization should hire women employees |  |  |  |  |  |
| ***Retaining diversity*** | | | | | |
| 1. Development opportunities for minority employees |  |  |  |  |  |
| 1. Increased representation of socially disadvantaged(disabled, poor, impaired, poor health) employees |  |  |  |  |  |
| 1. Development opportunities for women employees |  |  |  |  |  |
| 1. Organization should retain minority Employees |  |  |  |  |  |
| 1. Organization should retain socially disadvantaged employees |  |  |  |  |  |
| 1. Organization should retain disabled employees |  |  |  |  |  |
| 1. Development opportunities for socially disadvantaged(disabled, poor, impaired, poor health) employees |  |  |  |  |  |

***Part II:*** The following items are set to measure occupational work ethics specific to target firms. *The replies should range from* ***1: Strongly disagree to 5: Strongly agree.*** *This instrument has been adopted from the work of* ***Park & Hill (2017).***

| ***Item*** | ***1*** | ***2*** | ***3*** | ***4*** | ***5*** |
| --- | --- | --- | --- | --- | --- |
| ***Interpersonal Skill*** |  |  |  |  |  |
| 1. I like to be with other people. |  |  |  |  |  |
| 1. I am a happy person |  |  |  |  |  |
| 1. People like me. |  |  |  |  |  |
| 1. I use words like excuse me, sorry or please in my communication. |  |  |  |  |  |
| ***Initiative*** |  |  |  |  |  |
| 1. I do more than is required or expected of me. |  |  |  |  |  |
| 1. I am aware of what is going on around me. |  |  |  |  |  |
| 1. I accomplish my goals. |  |  |  |  |  |
| 1. I am eager to be successful. |  |  |  |  |  |
| 1. It is not difficult for me to find solutions to problems on my own. |  |  |  |  |  |
| ***Dependability*** |  |  |  |  |  |
| 1. I am good at following instructions. |  |  |  |  |  |
| 1. I follow the rules even if I disagree with them. |  |  |  |  |  |
| 1. I did not ever disappoint people. |  |  |  |  |  |

***Part III: T****he following items are set to measure Perceived organizational Performance (PoP).* ***The replies should range from 1: Strongly disagree to 5: Strongly agree.*** *This instrument has been adopted from the work of* ***Kundu and Mor (2017).***

| ***Item*** | | ***1*** | | ***2*** | | ***3*** | | ***4*** | | ***5*** | |
| --- | --- | --- | --- | --- | --- | --- | --- | --- | --- | --- | --- |
| 1. Market share highly booms for the firm |  | |  | |  | |  | |  | |  |
| 1. Productivity is at good stage |  | |  | |  | |  | |  | |  |
| 1. Return on Equity is improving |  | |  | |  | |  | |  | |  |
| 1. The company has appealing Profitability |  | |  | |  | |  | |  | |  |
| 1. The Customer service provision is alluring |  | |  | |  | |  | |  | |  |
| 1. Quality of products is appealing |  | |  | |  | |  | |  | |  |
