# Supplementary material for: The effect of workforce diversity on organizational performance with the mediation role of workplace ethics: Empirical evidence from food and beverage industry
Source: PLoS One. 2024 Jul 18;19(7):e0297765. doi: 10.1371/journal.pone.0297765 (PMC11257269; doi:10.1371/journal.pone.0297765)
Supplement: Attachment — Submitted filename: Rebuttal Letter.docx [file pone.0297765.s002.docx]

**REBUTAL LETTER**

**December 15, 2023**

**Dear Academic editor(Chunyu Zhang),**

The authors would like to thank the reviewers for their fair reviews and detailed list of constructive suggestions. We greatly appreciate the opportunity to submit a major revision of our paper for your renewed consideration. Below we respond to reviewers’ comments and point to the corresponding changes we made in the paper. Moreover, we have included the changes we made based on your (academic editor’s) directions/suggestions.

Kindly, consider the table below which clearly marks the comments forwarded and the changes made by the authors:

| **PLOS ONE Academic Editor(Chunyu Zhang)** | | |
| --- | --- | --- |
| **S.NO** | **Comments** | **Our responses/Adjustments** |
| 1. | Please ensure that your manuscript meets PLOS ONE's style requirements, including those for file naming. | Now I have used “PLOS ONE” referencing style available in Mendley.  All figures and tables are adjusted according to the standard guidelines of PLOS ONE. |
| 2. | Could you please provide further details on why your study is exempt from the need for approval and confirmation from your institutional review board or research ethics committee | Now, a letter illustrating ethical exemption of this study is included as sup file in the revised submission. |
| 3. | Please complete your Competing Interests on the online submission form to state any Competing Interests. If you have no competing interests, please state "The authors have declared that no competing interests exist." | This is made available in the cover letter. You can update the same on the submission system. |
| 4. | In your Data Availability statement, you have not specified where the minimal data set underlying the results described in your manuscript can be found. | The data is made available via Harvard Data verse and the URL is included in the cover letter. |
| 5. | Please ensure that you have an ORCID iD and that it is validated in Editorial Manager. | My ORCID 0000-0002-2708-1336 is validated and linked to editorial manager. |
| 6. | Please amend the manuscript submission data (via Edit Submission) to include authors Dr. Zerihun Ayenew and Dr. Gemechu Nemera. | These two co-authors are now added in the submission sytem. |
| 7. | Please clarify the number of Tables uploaded in your PDF file. |  |

| **Reviewer 1** | | |
| --- | --- | --- |
|  | **Comments** | **Our responses/Adjustments** |
| 1. | Abstract and Focus: The abstract should concentrate on findings that directly align with your research objectives, namely the impact of workforce diversity on performance and the mediating role of workplace ethics. Therefore, the discussion of these variable relationships is crucial. Devoting extensive space to the results of Confirmatory Factor Analysis (CFA) in the abstract detracts from these essential elements. | Technical details of CFA is removed from abstract and further demonstrations of findings are added. |
| 2. | Keywords Selection: The current keywords do not adequately capture the essence of your research. A thoughtful reconsideration to select keywords that more accurately reflect your study’s focus is recommended. | - The keywords are changed and inclusion and SEM are added in the new set. Most of the keywords have large word count in the manuscript which helps better in the articles discoverability. |
| 3. | Introduction Coherence: The introduction appears disjointed, with insufficient linkage between paragraphs. Each paragraph is too brief and scattered, lacking a cohesive narrative. Importantly, a clear delineation of the research's contribution and the knowledge gap it addresses is absent. | - Efforts has been made to maintain coherence among the paragraphs. - What is known and unknown is clearly marked - What is going to be addressed is clearly illustrated at the end of the introduction section |
| 4. | Clarification on EEO and Diversity: The relationship between Equal Employment Opportunity (EEO) and diversity needs to be more explicitly articulated. As it stands, the connection of this discussion to your main theme is unclear. | - The relationship between EEO and Diversity is vividly articulated. Besides their contradictions is also incorporated in the introduction section. |
| 5. | Methodology - Measurement of Diversity: In the methodology section, particularly regarding the measurement of diversity, clarify what PD & RD represent. Also, ensure the three numerical values mentioned are accurately represented and their significance is clear. | - The relevance of Cronbach’s alpha, Eigen value, and percentage variance explained are clearly illustrated in the revised manuscript. - Now PD and RD are written in an extended form. |
| 6. | Acronym Usage: Please provide the full name for OWEI-SF upon its first occurrence in the text to enhance clarity. | - The extended form of OWEI-SF is provided in the revised manuscript. |
| 7. | Clarification in Table 1: The meanings of 'nf' and 'ne' in Table 1 are unclear. An explanation is necessary for reader comprehension. | Explanations for nf and ne are provided below Table 1. |
| 8. | Citation for B-S Bootstrapping: It is imperative to include a citation supporting the use of B-S bootstrapping to correct data non-normality. | The statement is rephrased and three supportive citations are incorporated. Note that the table is transferred to appendix section labeled s Appendix Table1. |
| 9. | Structural Reorganization: Considerable reorganization of the manuscript is advisable. Large sections on EFA and CFA tests, which have limited direct relevance to your study, could be better placed in an appendix, with a focus on demonstrating the quality of your measurements. | - The EFA table, Convergent and Discriminant validity, Model Goodness of fit test of CFA are shifted in to appendix section. - PLOS ONE guideline urges the need to put tables immediately following their citation. Thus, though not most of the tables, I am able to transfer two of them (thought to be big size tables) to appendix section. |
| 10. | Relevance of SEM Comparisons: The rationale behind comparing models with and without control variables in Structural Equation Modeling (SEM), including different factor structures in CFA, does not seem directly linked to your research objectives. | - This part is removed in the revised manuscript with the notion that control variables does not make change or improvement on the findings. |

| **Reviewer 2** | | |
| --- | --- | --- |
|  | **Comments** | **Our responses/Adjustments** |
| 1. | Introduction and Literature review sections are weak and need rigorous work. For instance, introduction needs to be crafted in way that the problem exists in a society and research gap is there, based on the gap, identify the objectives of the study. . | - In the revised manuscript, introductions clearly indicate empirical knowledge gap and in what way does this study set to fill the gap. |
| 2. | As far as Literature is concerned, try to explain the variables in greater detail. | - The first section of the literature review briefly explains the variables of the study. |
| 3. | Why there is no support of theory in terms of hypotheses development? | - Thank you for bringing this in to our attention. Theories supporting the relationship between diversity and performance, diversity and ethics, and ethics and performance are incorporated in the revised manuscript. |
| 4. | In terms of Methods section, the context of Ethiopian beverages should be explained and Stratas must be further explained in detail. Minimum sample test should be conducted as well. | - The context of Ethiopian beverage is incorporated under the heading context, sample size and sampling technique. - The minimum sample test is incorporated with proper sample computation under the heading context, sample size and sampling technique. - All strata (A-H) are vividly and briefly explained. |

**Sincerely,**

**The authors**
