# Supplementary material for: The effect of workforce diversity on organizational performance with the mediation role of workplace ethics: Empirical evidence from food and beverage industry
Source: PLoS One. 2024 Jul 18;19(7):e0297765. doi: 10.1371/journal.pone.0297765 (PMC11257269; doi:10.1371/journal.pone.0297765)
Supplement: Attachment — Submitted filename: Rebuttal Letter.docx [file pone.0297765.s003.docx]

**REBUTAL LETTER**

**December April 14, 2024**

**Dear Academic editor (Chunyu Zhang),**

The authors would like to thank the reviewers for their fair reviews and detailed list of constructive suggestions. We greatly appreciate the opportunity to submit a revision of our paper for your renewed consideration. Below we respond to reviewer’s comments and point to the corresponding changes we made in the paper. Moreover, we have included the changes we made based on your (academic editor’s) directions/suggestions.

Kindly, consider the table below which clearly marks the comments forwarded and the changes made by the authors:

| **PLOS ONE Academic Editor(Chunyu Zhang)** | | |
| --- | --- | --- |
| **S.NO** | **Comments** | **Our responses/Adjustments** |
| 1. | Reviewer 3 suggests explaining the relationship between theory and model, which we think is very important. | Three paragraphs are added as an illustration for theoretical support, theoretically explaining the proposed relationships in the research model. Specifically, moral capital theory is incorporated in the manuscript as an overarching theory. Further, the same has been incorporated in the conclusion and implication sections as part of the description of theoretical implication. |

| **Reviewer 3** | | |
| --- | --- | --- |
|  | **Comments** | **Our responses/Adjustments** |
| 1. | Keywords should be in Alphabetical order. | - Thank you for the comment. Now all those six keywords are made in alphabetical order as you suggested. |
| 2. | Explain overarching theory and its connection with you research model. | - We kindly thank you for the comment! Because this fosters the quality of our manuscript. Thus, moral capital theory is introduced as an overarching theory to illustrate the existing logical relationships among the proposed dimensions in the proposed research model. The change is introduced just at the end of the review of related literature section before Fig. 1. |
| 3. | Conclusions and Implications must mention theoretical and practical implications as well. Further, Limitations and future directions for research may also increase the worth of manuscript. | - Limitations and future direction is introduced as one separate section in the manuscript that was not part of it but was just made following your comments. - Kindly we added two paragraphs to illustrate the theoretical and practical implications of the findings. |
| 4. | Cross check all the citations and references. Some of references do not have DOI address therefore download the citations from page of respective journals to include DOI. | - We have accessed the references, and their DOI has been incorporated into the revised version. For reports, books, conference proceedings, and other non-journal electronic documents that don’t have a DOI, we have introduced a URL. For some references where their url is long enough, we omitted the URL. |
| 5. | Attach data collection instrument (Questionnaire). | - Here we have attached the data collection instrument to this revised manuscript. Moreover, AMOS files and data,  both in Excel and SPSS formats, are available in a separate repository at Harvard Dataverse. It is made open and accessible, complying with the recently emerging open data policy of most journal publishers. The details are indicated in the methodology section. |

**Sincerely,**

**The authors**
