# Supplementary material for: The effect of workforce diversity on organizational performance with the mediation role of workplace ethics: Empirical evidence from food and beverage industry
Source: PLoS One. 2024 Jul 18;19(7):e0297765. doi: 10.1371/journal.pone.0297765 (PMC11257269; doi:10.1371/journal.pone.0297765)
Supplement: Attachment — Submitted filename: Rebuttal Letter.docx [file pone.0297765.s004.docx]

**REBUTAL LETTER**

**May 7, 2024**

**Dear Academic editor (Chunyu Zhang),**

No reviewer comments were forwarded in this review session but only a comment from you. Thus, only your request for an ethical approval letter is addressed.

Kindly, consider the table below which clearly marks the comments forwarded and the changes made by the authors:

| **PLOS ONE Academic Editor(Chunyu Zhang)** | | |
| --- | --- | --- |
| **S.NO** | **Comments** | **Our responses/Adjustments** |
| 1. | Please provide ethical approval. | An Ethical Approval letter from Arba Minch University is attached on ""Other"" file. |

**Sincerely,**

**The authors**
