# Supplementary material for: The effect of workforce diversity on organizational performance with the mediation role of workplace ethics: Empirical evidence from food and beverage industry
Source: PLoS One. 2024 Jul 18;19(7):e0297765. doi: 10.1371/journal.pone.0297765 (PMC11257269; doi:10.1371/journal.pone.0297765)
Supplement: Attachment — Submitted filename: Rebuttal Letter.docx [file pone.0297765.s005.docx]

**REBUTAL LETTER**

**June 29, 2024**

**Dear Academic editor (Chunyu Zhang),**

In this review session we have received constructive comments from Reviewer 5 and we noted from Academic editor’s comment about the need for resubmitting ethical clearance/approval letter. Thus, the comments forwarded from reviewer 5 and the issue raised by the academic editor are addressed.below.

Kindly, consider the table below which clearly marks the comments forwarded and the changes made by the authors:

| **PLOS ONE Academic Editor(Chunyu Zhang)** | | |
| --- | --- | --- |
| **S.NO** | **Comments** | **Our responses/Adjustments** |
| 1. | This study involves humans, and without providing ethical review evidence, this manuscript will not be published. | An Ethical Approval letter from Arba Minch University is attached on ""Other"" part of the submission system. We kindly request you to inform us if we have to use other way to submit it again. |

| **Reviewer 5** | | |
| --- | --- | --- |
| **S.NO** | **Comments** | **Our responses/Adjustments** |
| 1. | It would be beneficial to provide more details on the assumptions behind these analyses, particularly regarding the normality of data and the decision to use bootstrapping. Explaining how these assumptions were tested and met. | Thank you for bringing this in to our attention. Now in the revised manuscript the reason why we need to maintain normality else to use other robust technic such as bootstrapping is included with proper citation. |
| 2. | The manuscript discusses diversity in general terms. It would be valuable to delve deeper into specific dimensions of diversity (e.g., gender, age, ethnicity) and their unique impacts on organizational performance and workplace ethics. | We acknowledge this as constructive comment but we kindly apologize that the available data nodes are not sufficient to conduct recommended analysis because the study is designed and diversity related data were collected using general terms or subjective scales. Nonetheless, this valuable comment is incorporated as part of limitation of the study and set as direction for future studies to address. Directly your statement is quoted in limitation and future direction section of the revised manuscript. |
| 3. | The authors should elaborate on the practical implications of their findings for managers and HR professionals. Specific recommendations on how to effectively manage workforce diversity and enhance workplace ethics in the food and beverage industry would make the study more actionable. | Thank you!. A concluding remark has been added under conclusion and implication section of the manuscript showing directions how professionals and HR managers should address the issue in the food and beverage industry |
| 4. | While the study is comprehensive, it would benefit from a dedicated section on its limitations and suggestions for future research. Discussing potential limitations, such as the sample being limited to the Ethiopian food and beverage industry, and proposing areas for future studies can help contextualize the findings and encourage further investigation. | There is a separate section dedicated of showing future studies to perform similar studies in service sector where the scenario is quite different from food and beverage industry.  We have used the term “the sample being limited to the Ethiopian food and beverage industry” from your comments which we thought it is very important and illustrative. |
| 5. | Some sections of the manuscript, particularly the methodology and results, are dense and could benefit from clearer and more concise language. Breaking down complex statistical information into simpler terms and using visual aids (e.g., tables, figures) more effectively can enhance readability. | Thank you!The elongated illustrative statements regarding the thirteen strata in the methodology section and subsequent paragraph are revised and made brief and concise. Besides, the introductory paragraphs in results section are revisited to be concise as well. |
| 6. | The literature review is thorough, but integrating more recent studies and contrasting them with the current findings could provide a richer context. Highlighting gaps in the existing literature that this study addresses and discussing how the findings contribute to advancing knowledge in the field would be beneficial. | Thank you! This comment reinforces the manuscript and will make it more readable. Thus, four brief paragraphs are added before each proposed hypotheses illustrating the gaps detected on those recent studies and what this specific empirical study addresses.  This adjustment help us to add extra eight recent citations. |

**Sincerely,**

**The authors**
